# Diverging Mechanisms of Activation of Chemokine Receptors Revealed by Novel Chemokine Agonists

**DOI:** 10.1371/journal.pone.0027967

**Published:** 2011-12-09

**Authors:** Jose Sarmiento, Christie Shumate, Katsutoshi Suetomi, Aishwarya Ravindran, León Villegas, Krishna Rajarathnam, Javier Navarro

**Affiliations:** 1 Departments of Neuroscience and Cell Biology, The University of Texas Medical Branch, Galveston, Texas, United States of America; 2 Departments of Biochemistry and Molecular Biology, The University of Texas Medical Branch, Galveston, Texas, United States of America; 3 Sealy Centers for Structural Biology and Molecular Biophysics, and Molecular Medicine, The University of Texas Medical Branch, Galveston, Texas, United States of America; 4 Facultad de Medicina, Instituto de Fisiologia, Universidad Austral de Chile, Valdivia, Chile; 5 Multidisciplinary Pain Center, Aichi Medical University, Nagakute, Japan; 6 Department of Pharmaceutical Sciences, Universidad Peruana Cayetano Heredia, Lima, Peru; Emory University, United States of America

## Abstract

CXCL8/interleukin-8 is a pro-inflammatory chemokine that triggers pleiotropic responses, including inflammation, angiogenesis, wound healing and tumorigenesis. We engineered the first selective CXCR1 agonists on the basis of residue substitutions in the conserved ELR triad and CXC motif of CXCL8. Our data reveal that the molecular mechanisms of activation of CXCR1 and CXCR2 are distinct: the N-loop of CXCL8 is the major determinant for CXCR1 activation, whereas the N-terminus of CXCL8 (ELR and CXC) is essential for CXCR2 activation. We also found that activation of CXCR1 cross-desensitized CXCR2 responses in human neutrophils co-expressing both receptors, indicating that these novel CXCR1 agonists represent a new class of anti-inflammatory agents. Further, these selective CXCR1 agonists will aid at elucidating the functional significance of CXCR1 *in vivo* under pathophysiological conditions.

## Introduction

The onset of inflammation is mediated by the secretion of chemokines, which initiate the immigration of leukocytes from circulation to the site of injury and infection. The canonical chemokine CXCL8 (IL-8) binds with high affinity to two highly homologous chemokine receptors CXCR1 and CXCR2, which mediate pleiotropic responses including the onset of inflammation, angiogenesis, tumorogenesis and wound healing [1]. Chemokines are folded into three antiparallel β-sheets and a α helix on the top, with an unstructured N-terminus containing the ELR triad, and the CXC motif which connects the ELR to the N-loop and the 30 s loop [2]. The functional significance of CXCR1, the cross-talk between CXCR1 and CXCR2 in cells co-expressing both receptors, and their mechanisms of activation by CXCL8 and related ELR-CXC chemokines, are currently unknown. Whereas the functional role of CXCR2 can be examined by using CXCR2-deficient mice [3] or the administration of CXCR2 selective agonists (CXCL1-3 and CXCL5-7) or non-peptide CXCR2 antagonists [4], elucidating the role of CXCR1 *in vivo* is hampered by the lack of specific CXCR1 agonists and antagonists, and because mice and rat do not express CXCR1 in neutrophils and they do not express the human homologue of CXCL8 [5]. An argument is that CXCR1 is redundant; however the importance of CXCR1 was highlighted by studies suggesting that CXCR1 plays a significant role on the regulation and progression of chronic inflammatory disorders, including cystic fibrosis, COPD, and in sporadic urinary infections [6], [7].

On the basis of numerous mutagenesis and structural studies of chemokines and their cognate receptors, a two-site model was postulated for the interactions of chemokines with their cognate receptors [8]. Site **1** includes the receptor N-terminus, which recognizes the N-loop of chemokines, and site **2** includes extracellular loops of the receptor for binding to the N-terminus of chemokines to trigger receptor activation. This model, however, fails to account for the selective interaction of CXCR1 with CXCL8, and the non-selective interactions of CXCR2 with all ELR-CXC-chemokines, including those chemokines containing transplanted ELR triads into the nonELR-CXCL4 or the pseudo ELR in MIF1α [9], [10]. In this work, we have engineered CXCL8 derivatives via modifications in its N-terminus (ELR and CXC) and discovered novel CXCR1 agonists, which have allowed identifying the major interactions between ELR-CXC chemokines with their cognate receptors and probing the functional cross-talk between CXCR1 and CXCR2 in human neutrophils co-expressing both receptors.

## Results

### Engineering of CXCR1 Agonists

**Figure 1:**
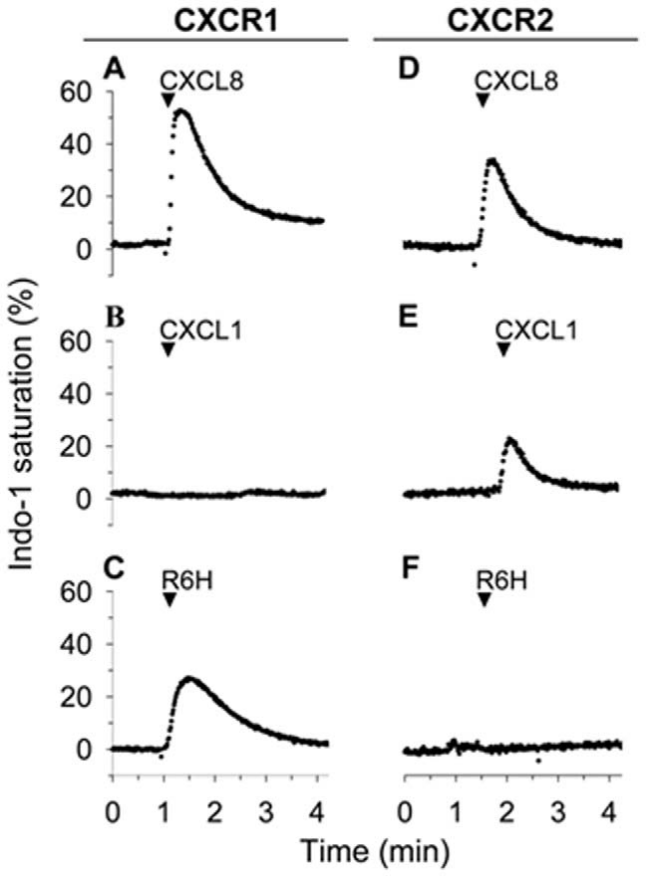
The R6H-CXCL8 mutant triggered calcium responses in HL-60 cells expressing CXCR1. HL-60 cells expressing CXCR1 or CXCR2 loaded with Indo-1 were stimulated with 100 nM of CXCL8, CXCL1 or R6H-CXCL8 mutant. The percentages of intracellular calcium responses are estimated from the calcium signal elicited by permeabilization of the cells with the detergent dodecylmaltoside (referred as 100%). The record is representative of at least five independent experiments.

To identify the sites in CXCL8 responsible for binding to CXCR e engineered CXCL8 derivatives by residue substitutions or deletions in the N-terminus of CXCL8 spanning the ELR triad and CXC motif. We found that CXCL8 mutants with residue substitutions of Arg6 in the ELR triad failed to increase the intracellular calcium in HL-60 cells expressing CXCR2 (**Fig. 1**) or in Jurkat cells expressing receptors for CXC or CC chemokines (data not shown). However, most of these CXCL8 mutants activated CXCR1, as demonstrated by the increase in intracellular calcium in HL-60 cells expressing CXCR1, according to the following agonist ranking His>Gly>Lys>Ala (**Fig. 1**, **Fig S1**). In contrast the CXCL8 mutant with Asp substitution in the Glu4 site of the ELR motif triggered weaker calcium responses than wild-type CXCL8 in HL-60 cells expressing CXCR1 or CXCR2 (data not shown), suggesting that Glu4 site is not important for the selective activation of receptor subtypes.

**Figure 2:**
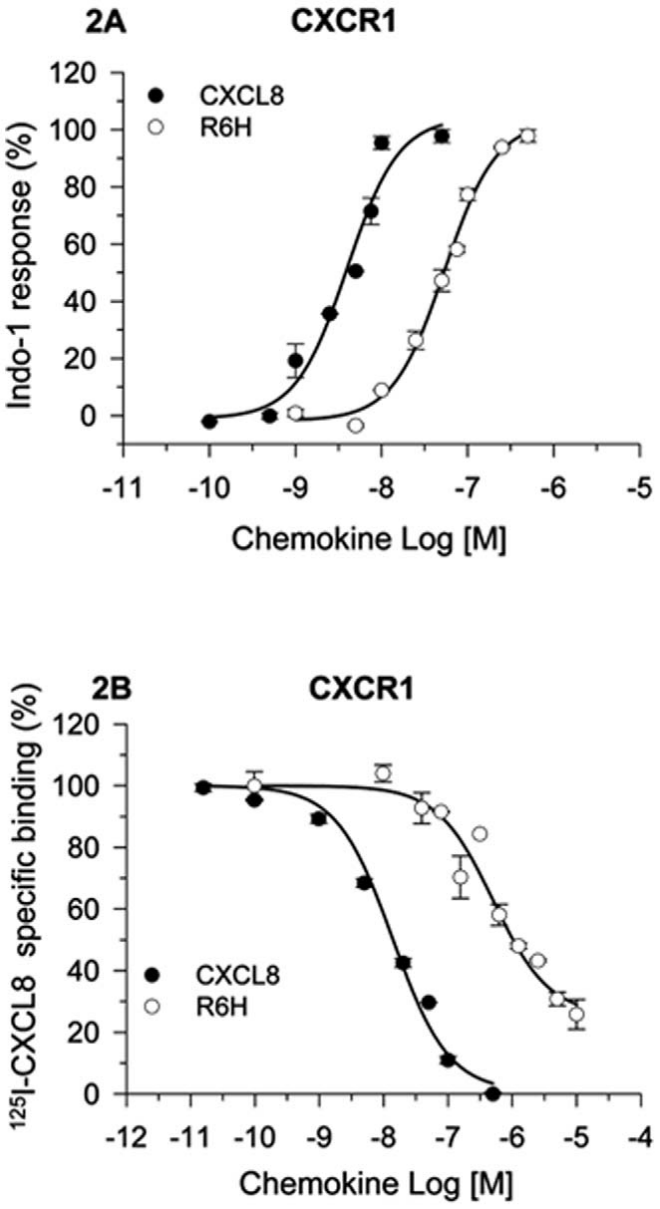
R6H-CXCL8 is a full CXCR1 agonist and binds to HL60 cells expressing CXCR1. **A.** Dose-dependent calcium responses in HL-60 cells expressing CXCR1. HL-60 cells expressing CXCR1 loaded with Indo-1 were stimulated with different concentrations of wild-type CXCL8 or R6H-CXCL8 mutant. The intracellular calcium response stimulated by 100 nM CXCL8 is referred as 100%. The EC_50s_ of R6H-CXCL8 and CXCL8 were 54 nM and 4 nM, respectively. Values are means of triplicate determinations, and the *bars* of each point represent the standard errors **B.** HL-60 cells expressing CXCR1 or CXCR2 were incubated with ^125^I-CXCL8 (0.16 nM) in the absence or presence of increasing concentrations of unlabeled CXCL8 or R6H-CXCL8 mutant at 4°C for 5 h. The 100% specific binding corresponded to the binding of ^125^I-CXCL8 in the absence of unlabeled chemokine minus the binding of ^125^I-CXCL8 in the presence of 200 nM of unlabeled CXCL8. The IC50s for R6H-CXCL8 and CXCL8 were 504 and 14 nM, respectively. Values are means of triplicate determinations, and the *bars* of each point represent the standard errors.

**Figure 3:**
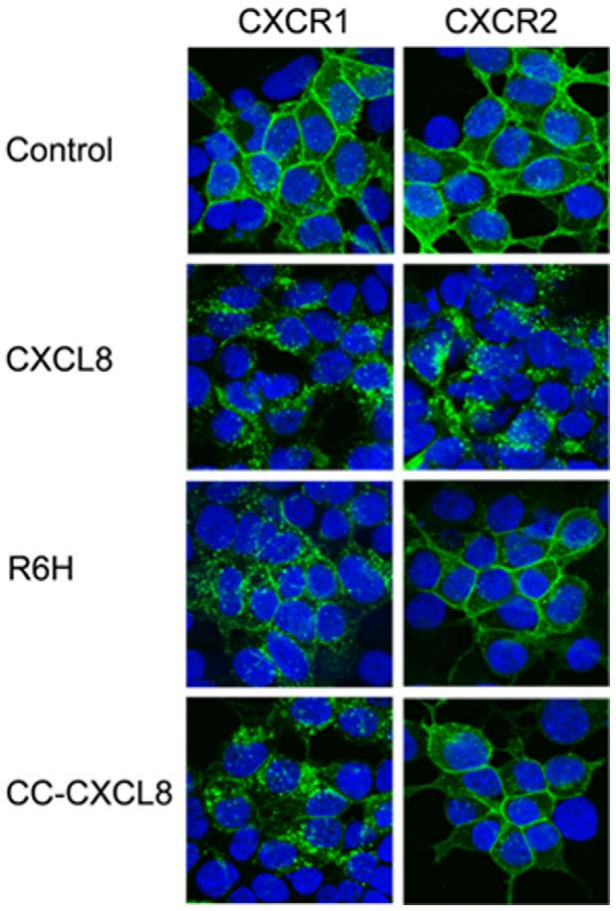
R6H-CXCL8 and CC-CXCL8 mutants triggered internalization of CXCR1. HEK 293 cells were transiently transfected with plasmids encoding CXCR1 or CXCR2 fused to GFP. Transfected cells were treated with 100 nM of CXCL8, R6H-CXCL8 or CC-CXCL8 mutants for 30 min at 37°C. Fluorescence was recorded by using a confocal microscope.

Dose-response analysis showed that the CXCL8 derivative (R6H-CXCL8) was a full CXCR1 agonist for calcium responses, although with a 13-fold higher EC_50_ than wild-type CXCL8 (**Fig. 2A**); moreover this mutant displaced the ^125^I-CXCL8 bound to CXCR1 but did not displace ^125^I-CXCL8- or ^125^I-CXCL1-bound to HL60 cells expressing CXCR2 (**Fig. 2B**, **Fig S2**). Similarly, the single residue deletion CXCL8 mutant CC-CXCL8 (CC replaced CXC) is also a selective full CXCR1 agonist but with a 100-fold higher EC50 than wild-type CXCL8 (**Fig S3**), as we previously described [11]. Accordingly, both R6H-CXCL8 and CC-CXCL8 triggered internalization of CXCR1 but not of CXCR2 (**Fig. 3**). To demonstrate the inflammatory activity of R6H-CXCL8 and CC-CXCL8 we used the rabbit model of skin inflammation, as rabbits like humans express CXCR1 and CXCR2 in neutrophils. Most importantly, rabbit express the human homologous of CXCL8, and rabbit CXCR1 and CXCR2 in neutrophils are functionally and pharmacologically similar to human CXCR1 and CXCR2 in neutrophils [12], [13]. Furthermore, R6H-CXCL8 did not displace the ^125^I-CXCL8 bound to CHO cells expressing rabbit CXCR2 (data not shown), suggesting that rabbit and human CXCR2 do not bind with high affinity to R6H-CXCL8. We found that that subcutaneous administration of R6H-CXCL8, CC-CXCL8, or CXCL8 in rabbits elicited skin inflammation (**Fig S4**), as demonstrated by the accumulation of neutrophils in the injection sites. These findings are in agreement with the failure of CC-CXCL8 to recruit neutrophils into mice lungs, as murine neutrophils do not express CXCR1 [11]. In contrast to the R6H-CXCL8 mutant, CC-CXCL8 binds with low affinity to CXCR2 (**Fig S2**). Interestingly, likewise to CXCR2 the decoy chemokine receptor DARC, which binds ELR-CXC and CCL2/CCL5 chemokines [14] failed to bind to R6H-CXCL8 or CC-CXCL8, as they did not displace the ^125^I-CXCL1 bound to DARC in ghost membranes from human red blood cells (**Fig S5**). These results showed for the first time the generation of selective CXCR1 agonists, but most importantly our data reveal that the N-terminus of CXCL8 contains the major determinants for the binding and activation of CXCR2 and binding to DARC, but not for CXCR1.

### Functional significance of CXCR1

**Figure 4:**
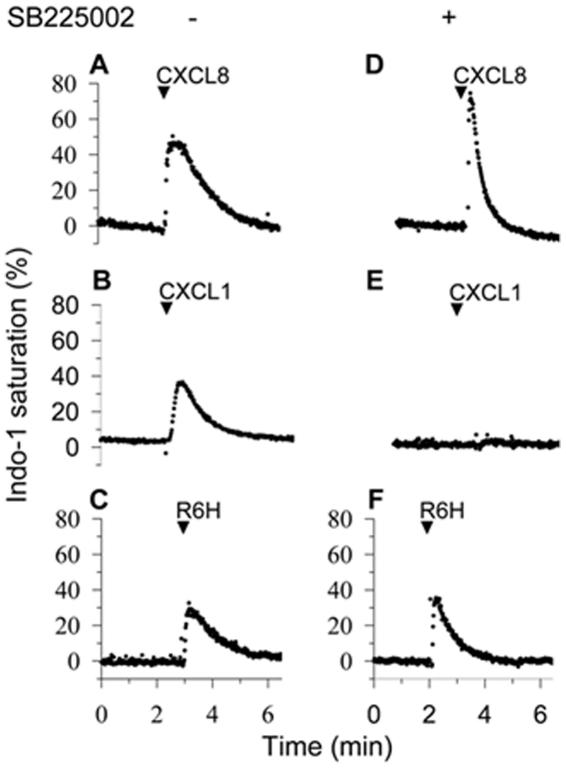
The R6H-CXCL8 mutant activated CXCR1 in neutrophils. Human neutrophils loaded with Indo-1 were treated with and without 1 µM of the CXCR2 inhibitor SB225002 and stimulated with 100 nM of CXCL8 (A and D), the CXCR2 agonist CXCL1 (B and E) and the R6H-CXCL8 mutant (C and F). The record is representative of at least five independent experiments.

The CXCR1 agonists identified in assays with HL-60 cells expressing CXCR1 or CXCR2 were probed in human neutrophils, which co-express both receptors. As expected we found that CXCL8 induced increase of intracellular calcium in neutrophils treated or untreated with the CXCR2 antagonist SB225002 [4] (**Fig. 4A and 4D**), as CXCL8 activates both CXCR1 and CXCR2. In contrast, the CXCR2 agonist CXCL1 did not induce calcium responses in neutrophils treated with SB225002 (**Fig. 4B and 4E**). Of importance, the CXCR1 agonists R6H-CXCL8 triggered calcium responses in neutrophils untreated or treated with SB225002 (**Fig. 4C and 4F**), further validating the selective activation of CXCR1 by R6H-CXCL8. Similarly, the CXCR1 agonists R6K, R6G and CC-CXCL8 also elicited calcium responses in neutrophils treated with SB225002, although at higher concentrations than R6H-CXCL8 or CXCL8 (**Fig S6**).

**Figure 5:**
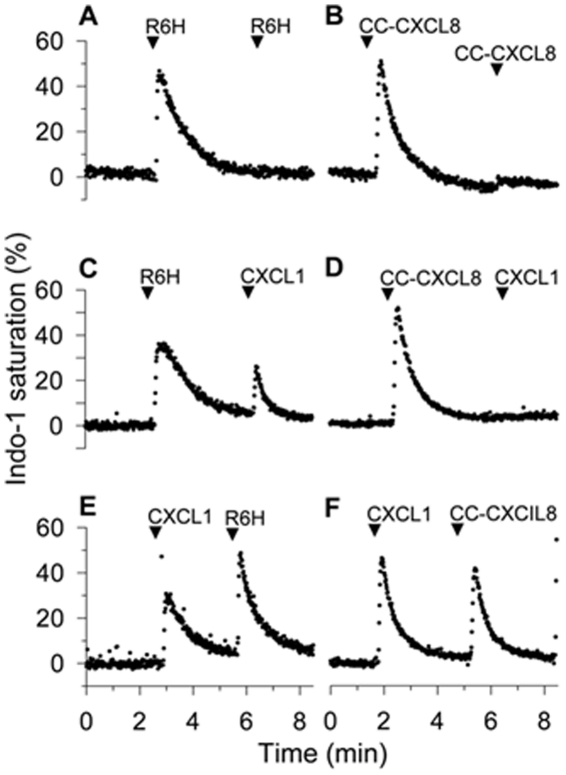
The R6H-CXCL8 and CC-CXCL8 mutants differentially desensitized the calcium responses mediated by CXCR2. Human neutrophils loaded with Indo-1 were first stimulated with 100 nM of R6H-CXCL8 and then challenged with 100 nM R6H-CXCL8 (A) or the 100 nM CXCR2 agonist CXCL1 (C). Similarly, neutrophils were first stimulated with 100 nM of CC-CXCL8 and then challenged with 100 nM CC-CXCL8 (B) or 100 nM CXCL1 (D). Conversely, neutrophils were first treated with 100 nM CXCL1 and then challenged with 100 nM of R6H-CXCL8 (E) or CC-CXCL8 (F).

To examine the functional interactions between CXCR1 and CXCR2 we probed the desensitization of calcium responses in neutrophils mediated by each receptor using the newly identified CXCR1 agonists (R6H-CXCL8 and CC-CXCL8). Both CXCR1 agonists R6H-CXCL8 and CC-CXCL8 desensitized the calcium responses mediated by the same agonists, as demonstrated by the lack of calcium response to a second agonist challenge (**Fig. 5A and 5B**). Interestingly, activation of CXCR1 by R6H-CXCL8 did not desensitized the calcium response to the CXCR2 agonist CXCL1, whereas CC-CXCL8 completely desensitized the responses to CXCL1 (**Fig. 5C and 5D**), indicating that these two CXCR1 agonists trigger distinct desensitization signals. As controls, CC-CXCL8 did not desensitize the CXCR2-mediated calcium responses in HL-60 cells expressing only CXCR2 (**Fig S7**), ruling out that the CC-CXCL8 desensitizes CXCR2 responses via the binding to CXCR2, as shown in **Fig S2**. Furthermore, activation of CXCR2 by CXCL1 did not desensitize the responses mediated by R6H-CXCL8 or CC-CXCL8 (**Fig. 5E and 5F**). Together, these results show that CXCR1 can elicit signals to desensitize calcium responses mediated by CXCR2 in a unidirectional fashion, as CXCR2 activation does not desensitize the CXCR1 responses.

### The CXCR1 N-terminus is the major determinant for binding selective CXCR1 agonists

**Figure 6:**
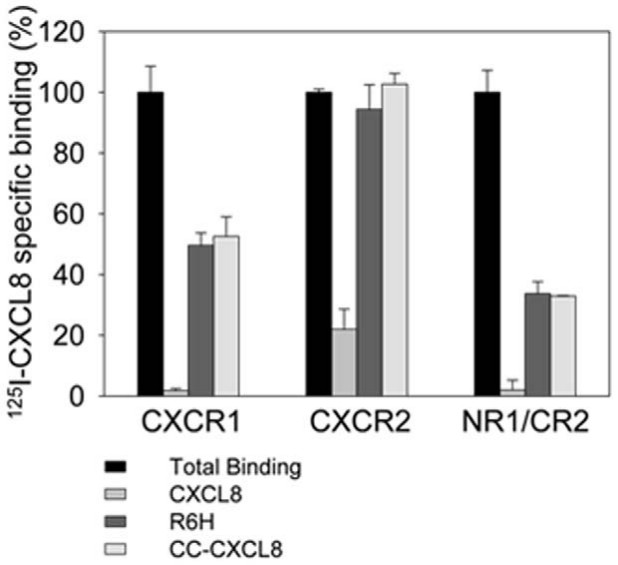
R6H-CXCL8 and CC-CXCL-8 mutants bound to the mutant CXCR2 containing the N-terminus of CXCR1. COS-7 cells transfected with plasmids encoding CXCR1, CXCR2 or the NR1/CR2 (chimera in which N-terminus of CXCR2 was replaced by N-terminus of CXCR1, as described [15] were incubated with ^125^I-CXCL8 (1 nM) in the absence (total binding) or presence of 100 nM unlabeled CXCL8 or 1 µM R6H-CXCL8 or CC-CXCL8. Values are means of triplicate determinations, and the *bars* of each point represent the standard errors.

We investigated whether the N-terminus of CXCR1 is essential for binding to R6H-CXCL8 and CC-CXCL8. We constructed a receptor chimera, in which the N-terminus of CXCR2 was replaced for the N-terminus of CXCR1. This chimera bound R6H-CXCL8 and CC-CXCL8 (**Fig. 6**). Interestingly this chimera failed to bind CXCL1. These findings are in good agreement with our previous studies with CXCR1/CXCR2 chimeras [15], in which the reciprocal exchange of N-termini switched the chemokine binding selectivity of the chimeric receptors. Moreover, we previously showed the important role of the CXCR1 N-terminus for binding to CXCL8 by transplanting the CXCR1 N-terminus into the mouse CXCR2, resulting in a mouse CXCR2 mutant exhibiting high affinity binding to CXCL8 [16].

## Discussion

These novel chemokine derivatives (R6H-CXCL8 and CC-CXCL8) represent the first generation of CXCR1 agonists, which will aid in probing the functional significance of CXCR1 *in vivo* under pathophysiological conditions, in particular in tissues co-expressing CXCR1, CXCR2 and DARC. In fact, we found that activation of human neutrophils, which co-express CXCR1 and CXCR2, with the newly engineered CXCR1 agonists desensitized the calcium responses mediated by CXCR2, but activation of CXCR2 did not desensitize the activation of CXCR1. This finding could have important implications in the regulation of inflammation, as CXCR1 agonists could operate as selective anti-inflammatory agents by preventing the activation of CXCR2, which is responsible for the accumulation of neutrophils into inflamed tissues due to injury or infection [4]. Engineering selective CXCR1 antagonists will complement the studies with the CXCR1 agonists in further defining the significance of CXCR1 in pathophysiological conditions.

**Figure 7:**
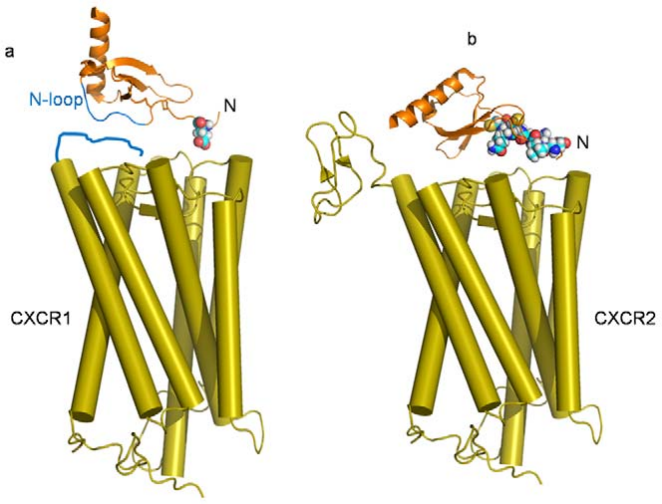
Models for the interaction of CXCL8 with CXCR1 and CXCR2. **a.** The N loop of CXCL8 (blue) binds to CXCR1 in site 1, which includes the N-terminus of CXCR1 (blue), the major determinant for the selective high affinity binding to CXCL8. Site 2 in CXCR1 binds Glu4 (sphere) of CXCL8 to trigger receptor activation. **b**. CXCR2 binds to the N-terminus of CXCL8 (Glu4, Arg6 and CXC are represented as spheres) to elicit receptor activation.

Importantly, these novel CXCR1 agonists are revealing for the first time the key structural elements in chemokines for the activation of CXCR1 and CXCR2, and compels to revise the current two-state model involving the interaction of chemokines with their cognate receptors. In this new model, the interactions of CXCL8 with CXCR1 and CXCR2 are different. On the basis of our data and previous studies [15], [16], [17], [18] CXCL8 binds to CXCR1 according to the classical two-site model (Fig. 7a), in which the N-loop of CXCL8 interacts with a receptor site (Site 1) defined by the N-terminus of CXCR1, a major determinant for the selective binding to CXCL8 to CXCR1. Site2 in CXCR1 binds to Glu4 of CXCL8 to trigger receptor activation without a major contribution from Arg6 or the CXC motif. On the other hand, we propose a new model for the interaction of CXCL8 with CXCR2, in which CXCR2 binds to the N-terminus of CXCL8 (Glu4, Arg6 and CXC motif) to trigger receptor activation, without significant contributions of either the N-terminus of the receptor or the N-loop of CXCL8 (Fig. 7b), as demonstrated by the broad binding of CXCR2 to all ELR-CXC chemokines containing non-conserved residues in their N-loops [19], and by the studies showing that the CXCR2 chimera containing the non-related N-terminus of the chemokine receptor CCR1 is still activated by all ELR-chemokines [17]. Finally, DARC is a hybrid of CXCR1/CXCR2, the conserved ELR-CXC in chemokines is required for binding to DARC, but the N-terminus of DARC is required for its binding to multiple chemokines (ELR-CXC and CC chemokines), as the CXCR2 mutant containing the DARC N-terminus exhibits the same binding profile as wild type DARC [14].

Optimization of our CXCR1 agonists will help designing more potent CXCR1 agonists and antagonists, which can be used as inflammatory modulators for the treatment of life threatening inflammatory disorders.

## Materials and Methods

### Expression and Purification of CXCL8 Mutants

cDNA encoding the human CXCL8 (1–66) were cloned into the *E.coli* expression vector pET32-Xa-Lic, which is used as template for engineering CXCL8 mutants by site directed mutagenesis. The wild type CXCL8 and mutants were expressed in *E.coli* BL21 (DE3) pLys and purified by chromatography fractionation using a His trap column, followed by a Mono S column or reverse phase column as described [11]. Alternatively, CXCL8 and mutants were also expressed in Insect S2 cells, which secrete CXCL8 mutants into the culture media. .

### Intracellular Ca^2+^ Mobilization

Human neutrophils were incubated with 5 µM Indo-1AM in Hank's solution without Ca^2+^ and Mg^2+^ at a density of ∼10^7^ cells/ml for 30 min at 37°C in the dark. HL60 cells expressing CXCR1 and CXCR2 were loaded as for neutrophils except that the cells were loaded with Indo-1 in RPMI. Subsequently, neutrophils and HL60 cells were washed with PBS and then resuspended in buffer containing 25 mM Hepes (pH 7.4), 125 mM NaCl, 5 mM KCl, 1 mM CaCl_2_, 0.5 mM MgCl_2_, 1 mM NaH_2_PO_4_, 0.1% bovine serum albumin, 0.1% glucose at a density of 10^7^ cells/ml. We employed 10^6^ cells/ml to record intracellular calcium in RF5301PC spectrofluorometer (Shimadzu), using an excitation wavelength of 330 nm and an emission wavelength of 405 nm, as described [20].

### ^125^I-CXCL8 and ^125^I-CXCL1 Binding

HL-60 cells (2×10^7^ cells/ml) expressing CXCR1 or CXCR2 or ghost membranes (75 ug of protein/ml) from human red blood cells were incubated at 4°C for 4–6 h in PBS supplemented with 0.1% (w/v) bovine serum albumin and 20 mM HEPES (pH 7.4), ^125^I-CXCL8 and ^125^I-CXCL1, in the absence and presence of unlabeled CXCL8 or CXCL8 mutants. The binding reaction for HL60 cells was terminated by centrifugation at 200×g for 5 min over a 10% sucrose cushion. The binding reaction for ghost membranes was terminated as for binding to HL60 cells, except that the centrifugation was carried out at 13,000×g for 5 min. The cell or membrane pellets were counted in a γ-counter.

#### Internalization of CXCR1 and CXCR2

cDNA encoding CXCR1 or CXCR2 were cloned in frame to EGFP in the mammalian expression vector pEGFP-N1. HEK 293 cells (150,000 cells) grown in glass cover slips coated with poly L-lysine (0.01%) were transiently transfected with the plasmids (1 µg DNA) encoding the chimeric receptors, by using Fugene-6. After 48 h the transfected cells were treated with CXCL8 or CXCL8 mutants for 30 min at 37C, then the cells were fixed with methanol at −20C for 45 min and stained with DAPI. The fluorescence was recorded by using a confocal microscope (Bio Rad Radiance 2100).

#### Expression of CXCR1 and CXCR2

HL-60 expressing CXCR1 and CXCR2 were engineered as described [11].The NR1/CR2 mutant cDNA encoded the transplantation of the N-terminus of CXCR1 into CXCR2 was constructed according to [15]. COS-7 were transiently transfected with the following constructs, CXCR1, CXCR2 and NR1/CR2 cloned into the expression vector pSVL using Fugene 6. After 48 hours the cells were harvested and bound to ^125^I-CXCL8 in the presence and absence of unlabeled CXCL8 wild-type and mutants, as described [15].

#### Rabbit skin inflammation

New Zealand rabbits (3–4 Kg) were maintained in the Animal Facility of the Universidad Peruana Cayetano Heredia (UPCH). Chemokine derivatives were injected into the shaved dorsal skin of rabbits, as described [21].All surgery was performed under sodium pentobarbital anesthesia, and all efforts were made to minimize suffering. This study was carried out in strict accordance with the recommendations in the Guide for the Care and Use of Laboratory Animals of the National Institutes of Health. The protocol was approved by the Institutional Ethics Committee for Animal Use (CIAE) of the Universidad Peruana Cayetano Heredia (Permit SIDISI 58343).

## Supporting Information

Figure S1 **The mutants R6X-CXCL8 triggered calcium responses in HL-60 cells expressing CXCR1. **HL-60 cells expressing CXCR1 or CXCR2 loaded with Indo-1 were stimulated with 1 µM R6X-CXCL8 mutants. The percentages of intracellular calcium responses are estimated from the calcium signal elicited by permeabilization of the cells with the detergent dodecylmaltoside (referred as 100%). The record is representative of at least five independent experiments.
(TIF)

Figure S2 **The mutant R6H-CXCL8 did not displace the ^125^I-CXCL1 or ^125^I-CXCL8 bound to HL-60 cells expressing CXCR2.** HL-60 cells expressing CXCR2 were incubated with ^125^I-CXCL1 (1 nM, **A**) or ^125^I-CXCL8 (0.16 nM, **B**) in the absence or presence of increasing concentrations of unlabeled CXCL1 or CXCL8 or R6H-CXCL8 or CC-CXCL8 at 4°C for 5 h. The 100% specific binding corresponded to the binding of ^125^I-CXCL1 in the absence of unlabeled chemokine minus the binding of ^125^I-CXCL1 in the presence of 200 nM of unlabeled CXCL1 (**A**). Similarly, the 100% specific binding corresponded to the binding of ^125^I-CXCL8 in the absence of unlabeled chemokine minus the binding of ^125^I-CXCL8 in the presence of 200 nM of unlabeled CXCL8 (**B**). Values are means of triplicate determinations, and the *bars* of each point represent the standard errors.
(TIF)

Figure S3 **Dose-dependent calcium responses in HL-60 cells expressing CXCR1.** HL-60 cells expressing CXCR1 loaded with Indo-1 were stimulated with different concentrations of wild-type CXCL8 or CC-CXCL8. The intracellular calcium response stimulated by 100 nM CXCL8 is referred as 100%. The EC_50s_ for CC-CXCL8 and CXCL8 were 316 nM and 4 nM, respectively. Values are means of triplicate determinations, and the *bars* of each point represent the standard errors.
(TIF)

Figure S4 **R6H-CXCL8 and CC-CXCL8 mutants induced skin inflammation in rabbits.** Prostaglandin E2 (PGE2, 30 nmol per 100 ul) was injected alone (control) or co-injected with 100 nmol of CXCL8, R6H-CXCL8 or CC-CXCL8 into the shaved dorsal skin of rabbits. After 3 h the animals were sacrificed and skin sections were stained with hematoxylin and eosin to identify neutrophil infiltration.
(TIF)

Figure S5 **R6H-CXCL8 and CC-CXCL8 mutants did not bind Duffy antigen (DARC).** Ghost membranes were incubated with ^125^I-CXCL1 (1 nM) in the absence or presence of increasing concentrations of unlabeled CXCL1, CXCL8, R6H-CXCL8 or CC-CXCL8 mutant at 4°C for 5 h. The 100% specific binding corresponded to the binding of ^125^I-CXCL1 in the absence of unlabeled chemokine minus the binding of ^125^I-CXCL1 in the presence of 200 nM of unlabeled CXCL1. The IC50s of CXCL1 and CXCL8 were 13.4 and 45 nM, respectively. Values are means of triplicate determinations, and the *bars* of each point represent the standard errors.
(TIF)

Figure S6 **The R6X-CXCL8 and CC-CXCL8 mutants triggered calcium responses in neutrophils. **Human neutrophils loaded with Indo-1 and treated or untreated with 1 µM SB225002 were stimulated with 1 µM R6X-CXCL8 mutants or 200 nM of CC-CXCL8. The percentages of intracellular calcium responses are estimated from the calcium signal elicited by permeabilization of the cells with the detergent dodecylmaltoside (referred as 100%). The record is representative of at least five independent experiments.
(TIF)

Figure S7 **CC-CXCL8 did not block the calcium responses mediated by CXCR2.** HL-60 cells expressing CXCR2 loaded with Indo-1 were first stimulated with 1 µM CC-CXCL8 mutant and then challenged with 100 nM CXCL1 and followed by a second dose of 100 nM CXCL1.
(TIF)

## References

[1] Sallusto F, Baggiolini M (2008). Chemokines and leukocyte traffic.. Nat Immunol.

[2] Clore GM, Gronenborn AM (1995). Three-dimensional structures of alpha and beta chemokines.. FASEB J.

[3] Milatovic S, Nanney LB, Yu Y, White JR, Richmond A (2003). Impaired healing of nitrogen mustard wounds in CXCR2 null mice.. Wound Repair Regen.

[4] White JR, Lee JM, Young PR, Hertzberg RP, Jurewicz AJ (1998). Identification of a potent, selective non-peptide CXCR2 antagonist that inhibits interleukin-8-induced neutrophil migration.. J Biol Chem.

[5] Lee J, Cacalano G, Camerato T, Toy K, Moore MW (1995). Chemokine binding and activities mediated by the mouse IL-8 receptor.. J Immunol.

[6] Hartl D, Latzin P, Hordijk P, Marcos V, Rudolph C (2007). Cleavage of CXCR1 on neutrophils disables bacterial killing in cystic fibrosis lung disease.. Nat Med.

[7] Ragnarsdottir B, Fischer H, Godaly G, Gronberg-Hernandez J, Gustafsson M (2008). TLR- and CXCR1-dependent innate immunity: insights into the genetics of urinary tract infections.. Eur J Clin Invest.

[8] Rajagopalan L, Rajarathnam K (2006). Structural basis of chemokine receptor function–a model for binding affinity and ligand selectivity.. Biosci Rep.

[9] Clark-Lewis I, Dewald B, Geiser T, Moser B, Baggiolini M (1993). Platelet factor 4 binds to interleukin 8 receptors and activates neutrophils when its N terminus is modified with Glu-Leu-Arg.. Proc Natl Acad Sci U S A.

[10] Kraemer S, Lue H, Zernecke A, Kapurniotu A, Andreetto E (2011). MIF-chemokine receptor interactions in atherogenesis are dependent on an N-loop-based 2-site binding mechanism.. FASEB J.

[11] Joseph PR, Sarmiento JM, Mishra AK, Das ST, Garofalo RP (2010). Probing the role of CXC motif in chemokine CXCL8 for high affinity binding and activation of CXCR1 and CXCR2 receptors.. J Biol Chem.

[12] Prado GN, Thomas KM, Suzuki H, LaRosa GJ, Wilkinson N (1994). Molecular characterization of a novel rabbit interleukin-8 receptor isotype.. J Biol Chem.

[13] Catusse J, Struyf S, Wuyts A, Weyler M, Loos T (2004). Rabbit neutrophil chemotactic protein (NCP) activates both CXCR1 and CXCR2 and is the functional homologue for human CXCL6.. Biochem Pharmacol.

[14] Lu ZH, Wang ZX, Horuk R, Hesselgesser J, Lou YC (1995). The promiscuous chemokine binding profile of the Duffy antigen/receptor for chemokines is primarily localized to sequences in the amino-terminal domain.. J Biol Chem.

[15] LaRosa GJ, Thomas KM, Kaufmann ME, Mark R, White M (1992). Amino terminus of the interleukin-8 receptor is a major determinant of receptor subtype specificity.. J Biol Chem.

[16] Suzuki H, Prado GN, Wilkinson N, Navarro J (1994). The N terminus of interleukin-8 (IL-8) receptor confers high affinity binding to human IL-8.. J Biol Chem.

[17] Ahuja SK, Lee JC, Murphy PM (1996). CXC chemokines bind to unique sets of selectivity determinants that can function independently and are broadly distributed on multiple domains of human interleukin-8 receptor B. Determinants of high affinity binding and receptor activation are distinct.. J Biol Chem.

[18] Lowman HB, Slagle PH, DeForge LE, Wirth CM, Gillece-Castro BL (1996). Exchanging interleukin-8 and melanoma growth-stimulating activity receptor binding specificities.. J Biol Chem.

[19] Baggiolini M (1998). Chemokines and leukocyte traffic.. Nature.

[20] Suetomi K, Lu Z, Heck T, Wood TG, Prusak DJ (1999). Differential mechanisms of recognition and activation of interleukin-8 receptor subtypes.. J Biol Chem.

[21] Forrest MJ, Jose PJ, Williams TJ (1986). Kinetics of the generation and action of chemical mediators in zymosan-induced inflammation of the rabbit peritoneal cavity.. Br J Pharmacol.

