# Correction: Diverging Mechanisms of Activation of Chemokine Receptors Revealed by Novel Chemokine Agonists

**DOI:** 10.1371/annotation/81d01b33-4eb2-459c-b600-0094d9f2d194

**Published:** 2012-05-24

**Authors:** Jose Sarmiento, Christie Shumate, Katsutoshi Suetomi, Aishwarya Ravindran, León Villegas, Krishna Rajarathnam, Javier Navarro

The funding statement should read: This work was supported by National Intitutes of Health grants GM081798 (J.N.), AI069152 (K.R.) and the University of Texas Medical Branch Claude D. Pepper Older Americans Independence Center National Institutes of Health/NIA Grant # P30 AG024832. The funders had no role in study design, data collection and analysis, decision to publish, or preparation of the manuscript. 

