# Supplementary figures and images for: Diverging Mechanisms of Activation of Chemokine Receptors Revealed by Novel Chemokine Agonists
Source: PLoS One. 2011 Dec 9;6(12):e27967. doi: 10.1371/journal.pone.0027967 (PMC3235101; doi:10.1371/journal.pone.0027967)

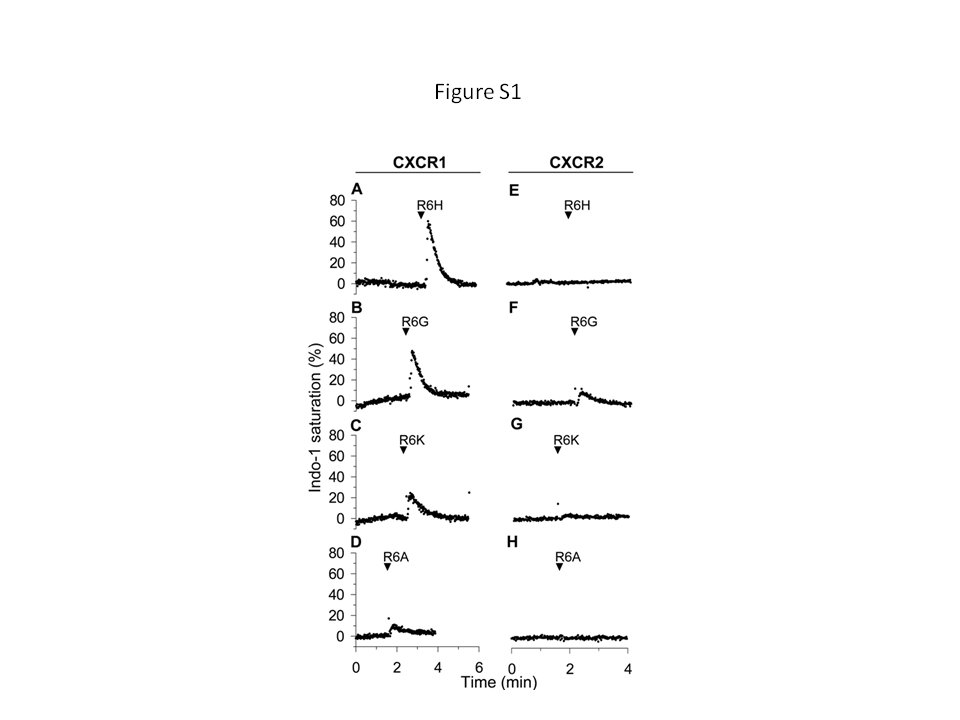

Supplement: Figure S1 — The mutants R6X-CXCL8 triggered calcium responses in HL-60 cells expressing CXCR1. HL-60 cells expressing CXCR1 or CXCR2 loaded with Indo-1 were stimulated with 1 µM R6X-CXCL8 mutants. The percentages of intracellular calcium responses are estimated from the calcium signal elicited by permeabilization of the cells with the detergent dodecylmaltoside (referred as 100%). The record is representative of at least five independent experiments. (TIF) [file pone.0027967.s001.tif]

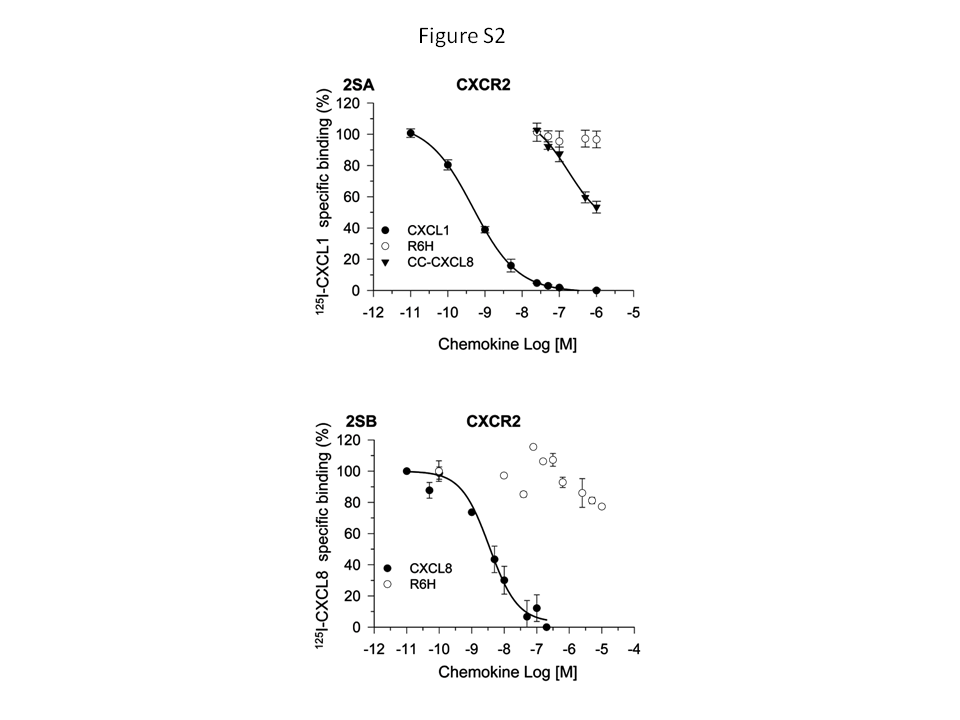

Supplement: Figure S2 — The mutant R6H-CXCL8 did not displace the 125I-CXCL1 or 125I-CXCL8 bound to HL-60 cells expressing CXCR2. HL-60 cells expressing CXCR2 were incubated with 125I-CXCL1 (1 nM, A) or 125I-CXCL8 (0.16 nM, B) in the absence or presence of increasing concentrations of unlabeled CXCL1 or CXCL8 or R6H-CXCL8 or CC-CXCL8 at 4°C for 5 h. The 100% specific binding corresponded to the binding of 125I-CXCL1 in the absence of unlabeled chemokine minus the binding of 125I-CXCL1 in the presence of 200 nM of unlabeled CXCL1 (A). Similarly, the 100% specific binding corresponded to the binding of 125I-CXCL8 in the absence of unlabeled chemokine minus the binding of 125I-CXCL8 in the presence of 200 nM of unlabeled CXCL8 (B). Values are means of triplicate determinations, and the bars of each point represent the standard errors. (TIF) [file pone.0027967.s002.tif]

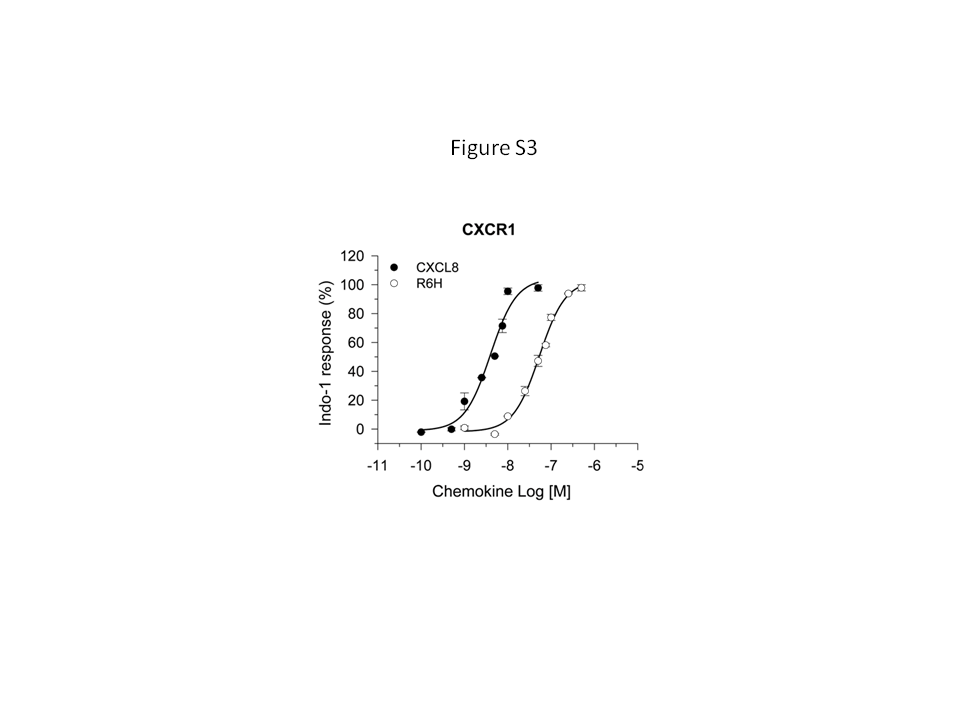

Supplement: Figure S3 — Dose-dependent calcium responses in HL-60 cells expressing CXCR1. HL-60 cells expressing CXCR1 loaded with Indo-1 were stimulated with different concentrations of wild-type CXCL8 or CC-CXCL8. The intracellular calcium response stimulated by 100 nM CXCL8 is referred as 100%. The EC50s for CC-CXCL8 and CXCL8 were 316 nM and 4 nM, respectively. Values are means of triplicate determinations, and the bars of each point represent the standard errors. (TIF) [file pone.0027967.s003.tif]

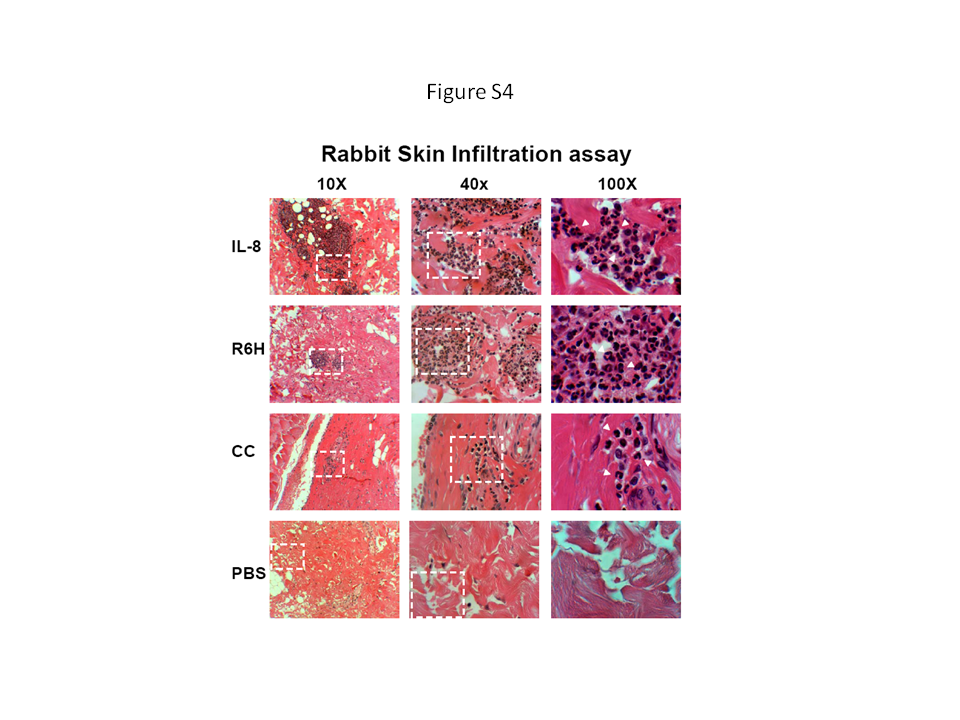

Supplement: Figure S4 — R6H-CXCL8 and CC-CXCL8 mutants induced skin inflammation in rabbits. Prostaglandin E2 (PGE2, 30 nmol per 100 ul) was injected alone (control) or co-injected with 100 nmol of CXCL8, R6H-CXCL8 or CC-CXCL8 into the shaved dorsal skin of rabbits. After 3 h the animals were sacrificed and skin sections were stained with hematoxylin and eosin to identify neutrophil infiltration. (TIF) [file pone.0027967.s004.tif]

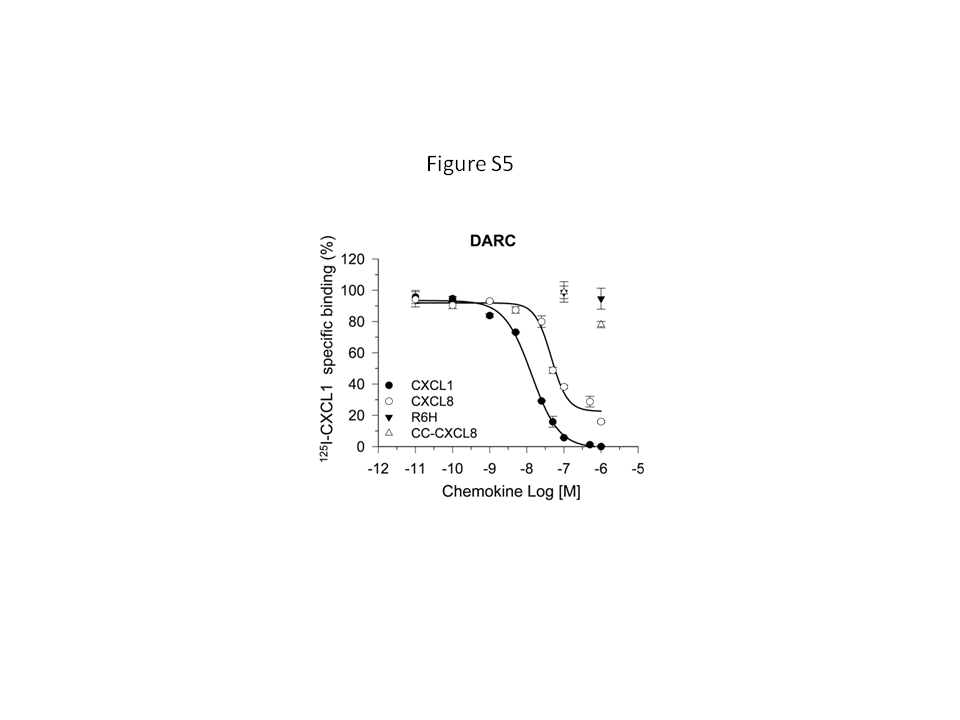

Supplement: Figure S5 — R6H-CXCL8 and CC-CXCL8 mutants did not bind Duffy antigen (DARC). Ghost membranes were incubated with 125I-CXCL1 (1 nM) in the absence or presence of increasing concentrations of unlabeled CXCL1, CXCL8, R6H-CXCL8 or CC-CXCL8 mutant at 4°C for 5 h. The 100% specific binding corresponded to the binding of 125I-CXCL1 in the absence of unlabeled chemokine minus the binding of 125I-CXCL1 in the presence of 200 nM of unlabeled CXCL1. The IC50s of CXCL1 and CXCL8 were 13.4 and 45 nM, respectively. Values are means of triplicate determinations, and the bars of each point represent the standard errors. (TIF) [file pone.0027967.s005.tif]

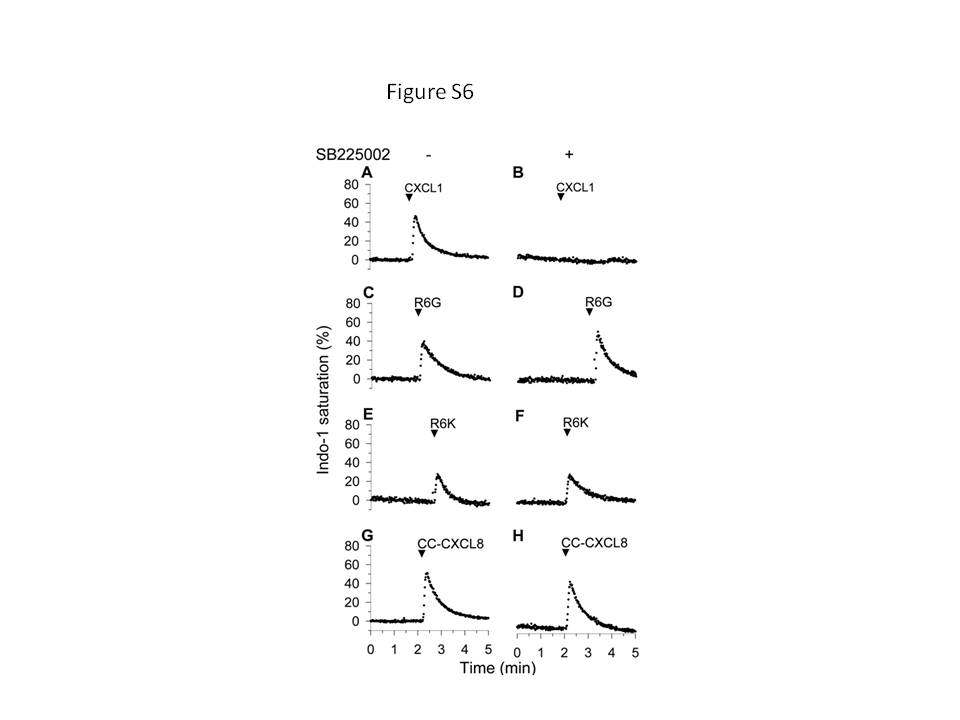

Supplement: Figure S6 — The R6X-CXCL8 and CC-CXCL8 mutants triggered calcium responses in neutrophils. Human neutrophils loaded with Indo-1 and treated or untreated with 1 µM SB225002 were stimulated with 1 µM R6X-CXCL8 mutants or 200 nM of CC-CXCL8. The percentages of intracellular calcium responses are estimated from the calcium signal elicited by permeabilization of the cells with the detergent dodecylmaltoside (referred as 100%). The record is representative of at least five independent experiments. (TIF) [file pone.0027967.s006.tif]

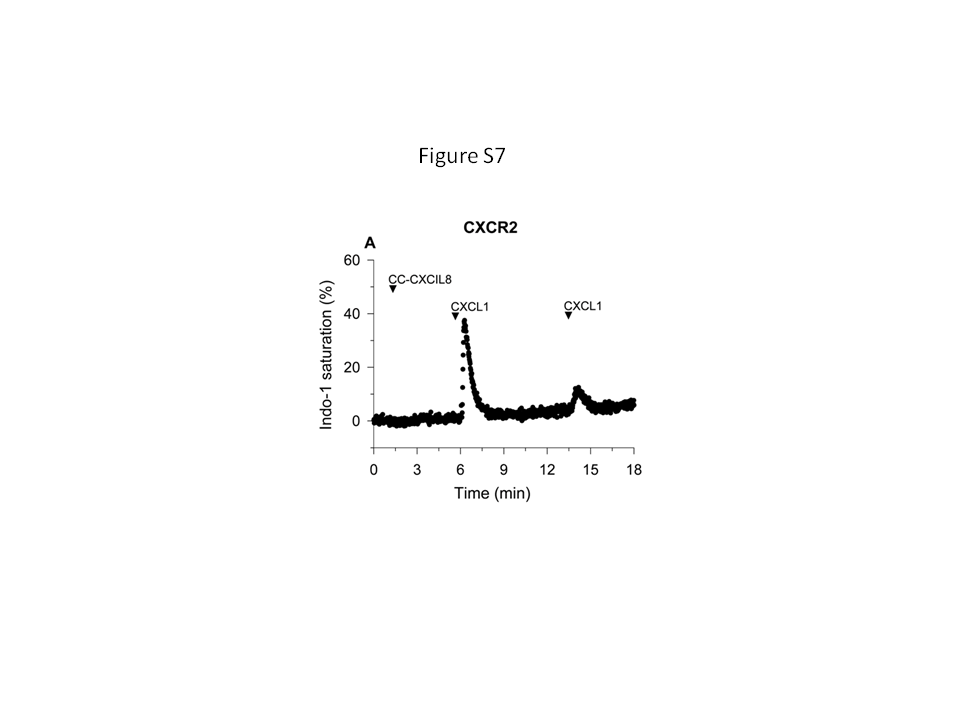

Supplement: Figure S7 — CC-CXCL8 did not block the calcium responses mediated by CXCR2. HL-60 cells expressing CXCR2 loaded with Indo-1 were first stimulated with 1 µM CC-CXCL8 mutant and then challenged with 100 nM CXCL1 and followed by a second dose of 100 nM CXCL1. (TIF) [file pone.0027967.s007.tif]
